# The ARV roll out and the disability grant: a South African dilemma?

**DOI:** 10.1186/1758-2652-15-6

**Published:** 2012-02-16

**Authors:** Marina Manuela de Paoli, Elizabeth Anne Mills, Arne Backer Grønningsæter

**Affiliations:** 1 Fafo, Institute for Applied International Studies, PO Box 2947, Tøyen NO-0608 Oslo, Norway; 2 Institute of Development Studies, Library Road, Brighton, BN1 9RE, United Kingdom; 3 Fafo, Institute for Labour and Social Research, PO Box 2947, Tøyen NO-0608 Oslo, Norway

## Abstract

**Background:**

Prior to the antiretroviral (ARV) drug roll out in 2004, people living with HIV (PLHIV) in South Africa received disability grants when they were defined as "AIDS-sick". In the absence of available and effective medication, a diagnosis of AIDS portended disability. The disability grant is a critical component of South Africa's social security system, and plays an important role in addressing poverty among PLHIV. Given the prevalence of unemployment and poverty, disability grants ensure access to essential resources, like food, for PLHIV. Following the ARV roll out in South Africa, PLHIV experienced improved health that, in turn, affected their grant eligibility. Our aim is to explore whether PLHIV reduced or stopped treatment to remain eligible for the disability grant from the perspectives of both PLHIV and their doctors.

**Methods:**

A mixed-methods design with concurrent triangulation was applied. We conducted: (1) in-depth semi-structured interviews with 29 PLHIV; (2) in-depth semi-structured interviews with eight medical doctors working in the public sector throughout the Cape Peninsula; (3) three focus group discussions with programme managers, stakeholders and community workers; and (4) a panel survey of 216 PLHIV receiving ARVs.

**Results:**

Unemployment and poverty were the primary concerns for PLHIV and the disability grant was viewed as a temporary way out of this vicious cycle. Although loss of the disability grant significantly affected the well-being of PLHIV, they did not discontinue ARVs. However, in a number of subtle ways, PLHIV "tipped the scales" to lower the CD4 count without stopping ARVs completely. Grant criteria were deemed ad hoc, and doctors struggled to balance economic and physical welfare when assessing eligibility.

**Conclusions:**

It is crucial to provide sustainable economic support in conjunction with ARVs in order to make "positive living" a reality for PLHIV. A chronic illness grant, a basic income grant or an unemployment grant could provide viable alternatives when the PLHIV are no longer eligible for a disability grant.

## Background

The estimated 5.7 million South Africans living with HIV in 2010 make this the largest HIV epidemic in the world [1]. HIV prevalence is highest among South Africa's majority black African population, which also has the highest rate of unemployment and the lowest per capita income of all the racial groups [2,3].

The roll out of antiretrovirals (ARVs) in the public health system started in 2004 in South Africa and followed a hard political struggle [4-6]. By 2010, more than 50% of those with CD4 counts lower than 200 cells/mm^3 ^received ARV therapy [1]. Although ARVs are fundamentally important in enabling people living with HIV (PLHIV) to live longer and healthier lives, it is crucial to understand the myriad of factors that constrain and shape life beyond the biomedical "problem/solution" framework that defines HIV as the problem and ARVs as the solution [7-10].

The South African approach to addressing poverty includes a broad range of measures from job creation to the establishment of basic services, such as housing, water and electricity, as well as welfare measures and social grants [11-13]. In this paper, we focus on the social grant system. At the end of 2008, the official unemployment rate among the black population was 25.9%, with unemployment in the townships even higher [14]. Despite high unemployment, South Africa's welfare system is based on the premise of full employment with no support system for the unemployed.

South Africa has one of the world's largest non-contributory social security systems [15,16]. Social grants are administered by the South African Social Security Agency (SASSA). It awards the following grants: old-age pension, disability, war veterans, care dependency, foster child, child support, grant-in-aid and social relief of distress [17]. In 2010, 14.3 million South Africans received social grants; old-age grants were awarded to 2.6 million South Africans, followed in number by disability grants, which were awarded to 1.2 million South Africans. The number of individuals receiving disability grants has more than doubled since 2000, rising to 1.4 million in 2008, and declining to 1.2 million recipients in 2010 [18]. The growth between 2000 and 2008 has been attributed to the expanding number of AIDS-sick people who were unable to access ARVs prior to the incremental roll out in 2004 [2,19].

The disability grant is a critical component of South Africa's social security system, and plays an important role in reducing poverty among PLHIV [20,21]. It provides financial assistance to people who are deemed "disabled" and therefore unable to seek or sustain employment. Disability grants are awarded either on a "temporary" basis (payments are provided for up to one year, whereupon the individual has to reapply for further support) or on a "permanent" basis (usually requiring renewal every five years). The Social Assistance Act of 2004 clarified the rules for awarding disability grants in general, but made no mention of HIV and AIDS. However, people who are sick with AIDS or ill with HIV-related opportunistic infections may be unable to look for and take up employment opportunities, in which case they qualify for a disability grant.

The disability grant aims at relieving the living conditions of people with disabilities and health constraints, but is also an important measure in the fight against poverty. The disability grant programme is designed to reach and assist households that have comparatively low incomes, more children, higher rates of adult unemployment and longer periods of exclusion from the labour force than non-recipient households [22]. The recipients generally belong to the black African population, which also tends to have lower levels of formal education. The disability grant programme absorbs those who are already excluded from the labour force. However, many needy recipients are not included in this programme [22].

The disability grant amounts to 1010 South African rands (US$1 = 7.38 ZAR; July 2010) (see http://www.sassa.gov.za/ABOUT-SOCIAL-GRANTS/GRANT-AMOUNT-652.aspx), which is roughly equivalent to the minimum wage for domestic workers in South Africa [23]. Prior to the roll out of ARVs in 2004, many PLHIV received disability grants. However, with better access to ARVs, many PLHIV stand to lose the grant as a result of their improved health. In combination with unemployment, the loss of the grant may increase food insecurity, which could have serious consequences for individuals on ARVs, given the need for proper nutrition to ensure treatment efficacy. These possibilities have led researchers to note that people may face trade-offs between adhering to their treatment and losing the disability grant when they become healthy versus not adhering to their treatment and continuing to remain eligible to receive the grant because they remain ill [2,19,24].

At the time of the study, the criteria used to assess disability varied from province to province; some provinces relied on assessments made by medical officers or district surgeons, while others convened "assessment panels" comprising social workers, medical doctors and "specialist" disability assessors from the Department of Social Development [22,25]. A general rule that applied in most hospitals and clinics throughout the country at the time when the study was conducted was that an individual with a CD4 count of ≤200 cells/mm^3^, roughly associated with clinical Stage 4 of AIDS, meets the clinical criteria for receiving a disability grant.

The aim of the study was to explore whether PLHIV experienced conflicts in choices involving their personal health and economic incentives with regard to the disability grant. Specifically, we investigated: whether the disability grant is an important source of income for PLHIV; whether PLHIV reduced or stopped treatment to remain eligible for the disability grant; and which factors doctors took into account when assessing whether PLHIV were eligible for a disability grant. In this paper, we explore this potential dilemma from the perspectives of PLHIV and doctors.

## Methods

In this study, mixed methods with a concurrent triangulation design were utilized [26]. A cross-sectional survey was conducted concurrently with qualitative in-depth interviews and focus group discussions (FGDs). Altogether, we conducted: (1) in-depth semi-structured interviews with 29 PLHIV; (2) in-depth semi-structured interviews with eight medical doctors working in the public sector throughout the Cape Peninsula; (3) three focus group discussions with programme managers, stakeholders and community workers; and (4) a panel survey, the Khayelitsha Select Panel Survey (KSPS), of 216 PLHIV receiving ARVs.

This combination of qualitative and quantitative data offered scope for rich comparisons. The quantitative and qualitative data were analyzed separately and integrated during the interpretation of results. This paper focuses on the qualitative findings, supplemented with quantitative figures from the KSPS. The quantitative sample was drawn from Khayelitsha township in Cape Town, while the qualitative study was conducted in four peri-urban townships (including Khayelitsha) in Cape Town in 2007-2008. The townships are within the boarder of the City of Cape Town Metropolitan Municipality. The focus of this paper is based on the qualitative findings, supported with quantitative data from the KSPS. The results from the quantitative findings have been published [27].

The first KSPS was conducted in 2004 with a baseline sample of 242 individuals receiving ARV treatment, and living in Khayelitsha, Cape Town. The survey was administered again in 2006 (n = 224) and in 2007 (n = 216). The survey collected data on a range of issues, including the impact of ARVs on economic activity, perceived and experienced HIV-related stigma, sexual behaviour, household composition, health-seeking behaviour and disclosure. Of special interest for this article are data about social grants, household economy and adherence to ARVs.

The in-depth interviews and focus group discussions were digitally recorded, transcribed and coded. The transcripts were carefully reviewed line by line, and codes were generated using the qualitative programme, Open Code [28]. The analysis set out to explore variations and common traits in the participants' narratives in order to gain a richer and more complete description of the themes addressed by the research questions. Drawing on the principles of grounded theory [29], external categories were not imposed on the data, but emerging and recurrent themes were identified from the data.

Informed consent was secured from each participant, and all names and identifying characteristics have been removed in order to ensure confidentiality. We use the term "participants" for those who participated in the in-depth interviews/focus group discussions and "respondents" for those who participated in the survey. The quantitative component of the study was approved by the Centre for Social Science Research's (CSSR's) Ethical Board. The City of Cape Town gave the researchers permission to conduct qualitative research on healthcare and social services in the public health sector.

## Results

### Ramifications of the disability grant for PLHIV

Although employment was desired by the HIV-positive participants, it was a reality for very few. In 2007, half of the KSPS sample (50%) was unemployed (Table 1); 71% of the households in the KSPS, were receiving a social grant as a part of their income, whereas 41% received a disability grant (Table 1). Most of the participants in the qualitative study were unemployed. They said that they struggled to maintain good health, as they could not afford to buy food. Sometimes hunger affected their adherence to ARV, as they could not take medication on an empty stomach. Further, the participants noted that it was important to adjust conditions of employment to accommodate the healthcare needs of PLHIV.

**Table 1:** Selected figures from the Khayelitsha Select Panel Survey, N = 216

	n	Percent
Unemployed	108	50

Receivers of disability grant	90	42

Respondents who had experienced losing the disability grant	90	42

Respondents who had stopped taking ARVs	6	3

Respondents who said they would stop taking ARVs in order to get sick so they might qualify for the disability grant again	0	0

Respondents who agreed that it is a common strategy for HIV-positive people to stop taking ARVs to get sick and qualify again for the disability grant	21	10

Households that received one or more social grant (all kinds of grants)	153	71

Source: Khayelitsha Select Panel Survey 2007 (KSPS)

A male participant highlighted the value of combining the provision of ARVs with employment in order to ensure "healthy living" for PLHIV:

"I am fine. I can work. The government must accept us (PLHIV) ... The government must produce more tablets ... We must get stronger ... We are not dead already! ... They must give us jobs, and they must treat us like anyone else (a person that is not HIV positive). They must not think that an HIV-positive person is going to die tomorrow. They are not giving us (PLHIV) opportunities to be somebody! ... So the government must not wait for somebody to die. The government must not wait until a person's CD4 count is less than 200, because that person cannot do anything! You understand? The government must look after and support PLHIV. I can get sick two, three times a week, but I do not want to be sick. I do want to get a job." (HIV-positive man, 44 years old)

This kind of sentiment was repeated throughout the narratives, particularly among the HIV-positive men. Another male participant felt the same kind of frustration with the government and spoke of the lack of employment opportunities in South Africa. He shared that in his household of nine people, only his retired mother received an income through her pension:

"The government is trying to make employment for everyone ... This HIV thing doesn't get cured, so the government must take care of its people. But the government can't afford to support us. My mother supports me now ... But at least with that grant, it was somewhat good. At least I could eat." (HIV-positive man, 50 years old)

Throughout the interviews, unemployment among PLHIV who had regained health from being sick with AIDS (through ARV medication) was a major theme. Further, the participants indicated that although they were not disabled, there were certain limitations to the kind of work they were able to do due to their HIV status, and to taking ARVs. For example, some participants said that they were reluctant to work night shifts, or to work outdoors, because they were more susceptible to contracting opportunistic infections due to the cold; night shifts also compromised their ability to adhere strictly to the ARV treatment regimen. In particular, participants noted that side effects from ARVs, like swollen feet, painful joints and nausea, limited the kind of work they were able to do as some people could not stand for long periods of time, or walk far distances to get to work.

The majority of the participants said that they were healthy and able bodied, but lamented the fact that they were unemployed because there was no available work:

"What makes me unhappy is the fact that I'm not working. If I can get a job, I will have a positive thinking. Now I am like somebody who is crippled, who is unable to do anything ... But I am healthy and willing to work! If you are working, you will have a positive thinking, and then you can focus." (HIV-positive man, 35 years old)

Most of the participants said that unemployment and poverty were their main concerns. At the same time, however, their HIV status was seen as an additional barrier to accessing the labour market, and as long as they were outside the labour market, they perceived managing their HIV status in a positive way - maintaining a healthy diet, for example - to be difficult. The disability grant was therefore viewed as a temporary way out of this vicious cycle of poverty and unemployment.

The majority of the grant was used to contribute towards general household expenses, and as such, it was sometimes the main or only source of income for both the HIV-positive individuals and members of their household. Of those receiving disability grants (KSPS), 98% used the grant to cover general (household) living expenses. The qualitative study also confirmed this finding, where general household expenses dominate the use of the grant money. With reference to the qualitative interviews, for most participants, the disability grant not only covers their individual and household living expenses, but also those of the extended family.

This need for and use of the disability grant is one of the factors putting people who were "granting" under pressure to ameliorate financial scarcity in their own households:

"The problem is that we have overcrowded families with huge numbers of children who are depending on one person who is "granting". My wife's and my own parents are both "granting", and it is the same situation. My mother is retired, but it is not easy for her to support me, because there are other people she lives with and they depend on her grant." (HIV-positive man, 35 years old)

Several of the PLHIV in the in-depth study indicated that they needed the grant in order to eat healthy food, which was seen as necessary for a person on ARVs. Furthermore, not having any food to eat was the main reason given for discontinuing ARV treatment, which will be further dealt with in next section.

### Disability grant termination and ARV adherence

Our findings indicate that the loss of income when disability grants are not renewed had a substantial impact on both the individual living with HIV and the household. However, the survey data did not indicate that PLHIV would choose poor health over grant loss. In the KSPS, not a single individual indicated that he/she would "stop taking ARVs" to "get (back) (his/her) disability grant" [27]. This finding was further supported by the qualitative findings from this study, although PLHIV certainly felt conflicted about the prospects of losing their disability grant.

One of the participants gave this description of what happens if the grant is not renewed:

"You become sick once you lose your grant, because you become depressed. After that, your CD4 count drops, because you don't know what you're going to eat. People become sick all the time because you need to take your medication. But, if you don't have food, it's difficult, and you cannot go back to your family. No one will help you. If you are unemployed, you will experience problems because you have to buy food and clothing and pay for funerals with that grant." (HIV-positive woman, 28 years old)

Some 42% of the KSPS respondents had experienced losing their disability grant. Ninety percent of those for whom the disability grant had been terminated, reported that it had a substantial impact on the household economy. The qualitative interviews clearly revealed that the loss of disability grants as a result of improved health had a significant impact on physical and emotional health and also led to high levels of stress. As the previous quote illustrates, losing the grant also has direct implications for PLHIV as a healthy diet may no longer be accessible, which in turn undermines adherence to ARVs. This was confirmed in all the interviews with the doctors who assessed disability grant eligibility and, as discussed shortly, placed pressure on doctors to ameliorate conditions of poverty through awarding the grant even when PLHIV did not strictly fall in to the grant eligibility criteria.

Discontinuation of ARVs in order to maintain and, in some cases, to re-qualify for the disability grant did not appear to be a common strategy. The actual ARV adherence rate was reported to be high in the KSPS; less than 3% of the respondents (n = 6) stipulated that they had stopped taking ARVs in 2007. However, in the qualitative research, each participant referred to "other" people who had stopped taking their ARVs in order to qualify for the grant and ameliorate their economic problems. The quantitative study supported this finding: 10.2% of the respondents in the KSPS agreed that "it is a common strategy for HIV-positive people to stop taking ARVs in order to get sick and get the disability grant back". One strong recurrent argument during the interviews was that if you had been severely ill from HIV and AIDS, you were not prepared to go back to a life of being sick in bed.

Thus, adherence to drugs in order to maintain health was important for the participants, as expressed by this male participant:

"Oh no, I take my drugs every day, because I do care about my treatment and all that since I started to take my treatment ... It's about my life!" (HIV-positive man, 44 years old)

The participants stressed that they would not consider undermining their health by failing to adhere to ARVs. That said, the qualitative interviews suggested a number of subtle ways in which PLHIV may "tip the scale" to lower the CD4 count without stopping ARVs completely. For example, increased alcohol consumption just before attending the clinic for a regular check up of the CD4 count was reported in the interviews. Similarly, some of the doctors reported that skipping some days of treatment in order to become slightly sick and reduce their CD4 counts was a strategy that PLHIV adopted to prolong the period of eligibility for the disability grant.

As indicated by this quotation, most doctors were of the opinion that their patients practised a form of "circumstantial" non-adherence:

"My patients don't take the tablets every day; they play with their health. The cleaners here at the clinic find lots of tablets dumped daily, in the yard, toilet. It is almost every day that one of my patients will tell me that they saw someone in the bathroom who dropped a handful of tablets in the toilet. Sometimes, I hear them when they discuss outside about manipulating the number of tablets. They calculate how many tablets they should be left with and they educate one another outside. They discuss among themselves that if you take your tablets every day, you will get better and your CD4 count will go up and that's when the grant will stop. So they play with their health." (Male doctor)

Some PLHIV confessed that they did not always take their drugs on a regular basis. The next quote highlights the complexity of securing health in the context of poverty and unemployment, particularly given the importance of food for ARV adherence:

"I just tell the doctors, sometimes, when I am hungry, I don't take my ARV treatment, I just leave the tablets lying there ... and then I'll see if I have any food to eat the following day." (HIV-positive woman, 30 years old)

The qualitative data confirmed that PLHIV would give priority to their health and risk losing the grant, but that it was difficult to take the drugs on an empty stomach. Unemployment, therefore, has practical consequences for the participants' experience of poverty and their ability to maintain good nutrition alongside their ARV regimens. During one interview, a male participant laughingly stated, in reference to his clinic:

"They keep pumping me with medication, so they must keep pumping me with food as well!" (HIV-positive man, 50 years old)

### Doctors' role and their relationship with PLHIV

There are a number of criteria for qualifying for a disability grant, including being between 18 and 59 years of age, holding South African citizenship or refugee status, and permanently residing in South Africa. These requirements apply across all social welfare grants in South Africa; the distinction in qualification criteria for disability grants lies in the medical assessment, in which a qualified medical professional must confirm the candidate's "disability" [14]. Medical doctors, therefore, have authority to decide whether or not PLHIV are eligible for a grant, and to stipulate the duration of the grant award. In light of the findings just outlined, which point to the interlinked nature of physical health with financial security, doctors play a pivotal role in shaping the physical and financial well-being of PLHIV.

Doctors are required to write a grant assessment in order for PLHIV to apply for disability grants; however, many doctors stated that the particularities of the assessment were unclear, and that the complex interplay of physical health with financial and psychological well-being made it even more difficult to base decisions on biomedical indicators of health like CD4 counts. The absence of a clear assessment framework gave them some flexibility in evaluating grant eligibility, but it also increased the pressure placed on doctors by HIV-positive clients. Some doctors acknowledged that in addition to health criteria, like CD4 counts, they also used social criteria when deciding who qualifies for a disability grant. Doctors reported feeling uncomfortable and pressured to recommend disability grants for PLHIV.

They said that expectations around the disability grant was the most important challenge facing their daily work:

"The most difficult thing about being a doctor is that you have to write disability grants. It is like you are God; you just have to look at the person's face and decide about whether they qualify or not." (Female doctor)

The participants echoed this sense that the doctors were like God, dictating the quality of life for an unemployed HIV patient. They also felt uncomfortable with the extent to which doctors shaped their lives. The participants in the qualitative study reported feeling helpless when confronted with the doctor's inherent power to make decisions that affected their financial and physical well-being. The absence of clear guidelines for recommending the disability grant reinforced the participants' perception that doctors made ad hoc decisions that were inconsistent, and dependent on subjective factors. When discussing the extension of the original disability grant time period, one participant stated:

"It depends of the heart of the doctor sometimes. If the doctor has got your sympathy, then he can do that." (HIV-positive woman, 28 years old)

In the survey, 51% of the respondents attributed the loss of their disability grants to their doctors' decisions that they were no longer eligible for the grant, and 29% stated that their applications were refused. Thus, the doctors' assessments emerge as the most important reason for terminating the grant from the perspectives of the patients. Before the era of universal access to ARVs, a permanent disability grant was provided to PLHIV who were in the final stage of AIDS. In our study, we found that doctors varied greatly in the criteria they used to assess eligibility and whether they prescribe "temporary" or "permanent" grants.

In the next quote, the doctor describes the way in which doctors reach decisions around disability grant eligibility:

"Nationally there are no clear guidelines. It varies from province to province, and even in this province, it varies from doctor to doctor. Some doctors are hesitant to write a disability grant while others give just about anyone a disability grant. Some doctors still prescribe permanent disability grants (duration five years), while other doctors never do it. Locally, there has been some sort of agreement, if your CD4 count is less than 200, then you qualify for a grant; however, some doctors will give it to you for six months and other doctors will give you a 12-month grant." (Female doctor)

As indicated, the rationale for prescribing grants differed between doctors, which reinforced the participants' belief that the decisions regarding disability grant eligibility and duration were ad hoc and inconsistent. For example, some employed patients with high CD4 counts were still receiving disability grants, while unemployed patients with low CD4 counts were unable to receive the grant.

Some of the doctors believed that some of their patients use HIV as a way to get grants. These doctors strongly believed that unemployment was the problem and that the measures should be focused on unemployment instead of disability. In their logic, a disability grant was the wrong policy response to the problems caused by unemployment. On the other hand, they were aware that the disability grant gave new opportunities to PLHIV in an everyday situation where it was hard to manage the economic demands that the household was facing.

This also puts doctors under pressure to balance the biomedical criteria for grant eligibility against other criteria, such as the individual's circumstances, unemployment and the poverty in which many HIV-positive South Africans live:

"I always enquire about their employment. If they are employed, and their general health condition is good, I do not offer [a] disability grant. In cases where there is no income through employment, I offer [a] disability grant. I have offered disability grant[s] to all those who start ARVs regardless of their CD4 count. I have even prescribed ARVs for patients whose CD4 count is above 200. I base my assessment for initiation of ARVs on the general health condition of the patient." (Female doctor)

Another doctor, however, was more reluctant to incorporate social dimensions into her assessment of an individual's disability grant eligibility:

"Sometimes they bring their kids and you can see that they are hungry. But then again, the guidelines state that you cannot give this person a disability grant. You know what is going on at home and that there are no social workers to take care of the person. You know that you can help, but then at the same time you do not want to be seen as the "fraudulent" doctor. I guard against that because one day when you give that disability grant and SASSA decided to do an audit - then you may be seen as fraudulent. But honestly speaking, I have been "fraudulent", two or even three times, not a lot; I mean I am very careful. They (HIV-positive patients) think I have empathy and understanding, because where they come from is probably where I come from." (Female doctor)

The participants in the in-depth interviews were unclear about the way the doctors decided on grant eligibility and the way the social services managed the grants. Some of the participants described their frustration with corruption in the welfare system, referring to officials working in the social security agency who demanded the first month of the disability grant payment as payment for them assisting in the disability grant application procedure. Some PLHIV clearly indicated that the application procedure was difficult and suggested that it would be a useful to have professional people assisting them in the process.

The narratives included accounts of people who had received a disability grant in the 1990s and were still accessing it even though their health was good due to ARV treatment. They reported that these people on "long-term grants" had received the grant from a friendly doctor at a time when some doctors interpreted the rules to mean that PLHIV without access to treatment would qualify for permanent grants. This was before the roll out of ARVs.

In most cases, grant recipients were not aware of how long they had the grant, and a few of the participants seemed to wonder why they still received the grant. One recurrent story was linked to people who got the grant for six months. We were told that they could not apply for a new grant period before the previous one had ended, and then it took some months before they got a new grant. As a result, the story of a person receiving a grant might sound like this: six months with a disability grant, then a period without a grant, and then another six months with a grant again, and so on. One of the in-depth participants, an HIV-positive 44-year-old man, stated, "The grant is coming and going."

## Discussion

South Africa's high level of unemployment complicates the development of a comprehensive welfare system based on income tax revenues [13,30]. We found high unemployment among both HIV-positive and HIV-negative individuals. We also found a high level of disability grant recipients among the participants living with HIV. A comparative study of households in the Free State Province of South Africa illustrates the impact of unemployment on HIV-affected households relative to unaffected households [31]. Affected households had incomes and expenditures that were 14% to 26% lower than non-affected households [31].

The two key narratives that emerged from the interviews suggested that unemployment affects PLHIV in two ways: HIV illness may prevent PLHIV from seeking work; and even if PLHIV are able to seek work, they are unlikely to find work given South Africa's high unemployment levels. The participants struggled with the dual impact of the loss of their disability grants as their health improved, and the general lack of employment opportunities. Similarly, Coetzee found that ARVs enabled PLHIV to look for work in the labour market, but given the high levels of unemployment, ARVs and resumed health did not translate directly into employment [32].

The number of grants awarded to HIV-positive individuals rose from 27% in 2001 to 41% in 2003 [33,34]. Between 2001 and 2004, this notable increase corresponds to the low initial level of the roll out of ARVs. Nattrass has suggested that the increased number of disability grants awarded to PLHIV during these years may reflect institutional changes that enabled decision makers to use the grant as a means of alleviating poverty [33].

Sustained adherence to ARVs is crucial to ensure the effectiveness of treatment and to slow the development of AIDS and the spread of drug-resistant viral strains. People who were taking ARVs had less functional impairment, fewer HIV-related symptoms, and a greater capacity to work than those who had not yet started ARV treatment [35].

Our findings indicate that the disability grant played an important role in minimizing the negative consequences of unemployment among PLHIV. The disability grants played a crucial role in maintaining their financial, emotional and physical well-being. However, we found a number of conflicting views as to whether disability grants were a disincentive to adherence. Despite the reported reliance on disability grants among PLHIV in the context of high unemployment levels, this study did not find that PLHIV taking ARVs sought to compromise their health in order to remain eligible for the grant. However, a few participants mentioned their own and other people's strategies for reducing their CD4 count prior to their tests through alcohol consumption and short-term discontinuation of their ARVs.

Even though the PLHIV and the doctors described the adherence to medication in quite different ways, they seemed to agree about the consequences of receiving or losing the grant. These findings suggest nuanced approaches to balancing health and welfare where health usually takes precedence. Alcohol abuse, insufficient financial resources and lack of food were also found to act as barriers to ARV adherence by Nachega and colleagues [35]. A systematic review of adherence studies yielded a pooled estimate of 77% adequate adherence in Africa, whereas in North America, the corresponding figure was 55% [36]. A meta-analysis found that barriers to adherence were consistent in multiple settings, for example, fear of disclosure, access to medication and work and family responsibilities [37].

Even though we did not find strong evidence that low adherence, coupled with the aim of re-qualifying for a disability grant, was a widespread phenomenon, we found that some PLHIV practised a form of "circumstantial" non-adherence. It was also clear that the grant application system puts PLHIV under pressure because of the uncertainty related to the renewal and loss of the disability grant. This in turn could result in a vicious cycle where PLHIV experience a restoration of health but have difficulty maintaining a nutritionally adequate diet after the loss of disability grant income when no other sources of income are available. A secure source of income is necessary to ensure a good diet for PLHIV, and this in turn is pivotal for adherence to the medication.

The most common reason for non-adherence given in the interviews and survey was hunger. This problem is linked to the fact that in some cases, the disability grant was the only source of economic relief for PLHIV. Coetzee and Nattrass noted that as ARVs restored the health of PLHIV, the unemployment rate increased because despite their improved health, many individuals were still unable to find employment [38].

In contrast to studies that present health and welfare as an oppositional dyad, this study offers scope for engaging with the subtleties of negotiating health and welfare in a country struggling with high rates of HIV prevalence and unemployment [39,40].

Disability grants are not a sufficient response to intransigent poverty. This puts pressure on both the state and local decision makers, like doctors, to find alternative means of supporting individuals and families affected by HIV and unemployment. Citing Fakir, Jones summarizes the challenges of providing social services in South Africa; these include institutional weaknesses, gaps in quality, and a failure to adequately focus on the norms, values and attitudes of public officials with regard to HIV [41,42]. In addition, there is a need for greater responsiveness and accountability. Problems in the service delivery system have led to protests that coincided with the protests against delayed ARV roll out.

Similarly, Macgregor discussed the ways in which the notion of health as a human right engenders expectations of the state to provide the means to ensure health [43]. She described how doctors in a psychiatric clinic made diagnoses and negotiated the grant for HIV-positive persons. We found that this role of the "kind" doctors, mediating between the system and the individual, entails achieving a balance between medical and social criteria when recommending disability grants. For the doctors, the expectations of their patients were a difficult challenge. Leclerc-Madlala called attention to the inadequacy of the social assistance system and to the importance of reducing poverty in the fight against HIV and AIDS [24]. She argued that it was important to understand and respect the social context of each PLHIV.

Doctors are deemed "fit" to make medical assessments for the grant-issuing authority (SASSA) so they play a critical role in the administration of the grants. Their role of assessing grant eligibility in the public health system is based on their integration into the public services. Given that the South African social welfare system places the responsibility for determining disability grant eligibility on doctors, our findings indicate that doctors experience considerable pressure when recommending eligibility, so that they use different kinds of criteria to determine eligibility.

Through their role in determining eligibility, doctors have a strong influence on each individual's quality of life. As long as the disability grant aims at compensating for a clinical condition or a disability, the doctors' role will remain pre-eminent. If the disability grant primarily becomes a means for poverty relief, the doctors' role becomes problematic. The crucial role of doctors in approving the grant applications combined with weak institutional structures and the needs of the PLHIV revealed in our study, may explain why the doctors feel that they are under such pressure. It is likely that the institutional weaknesses of the social services increase the pressure on the doctors by further enhancing the importance of their role in the system. The general lack of clarity with regards to the guidelines and the variation in the ways in which the grant is approved also contributes to the doctor's increased importance, and perhaps to the participants' perception of "inequity" in grant eligibility and administration. It seems that the pressure on the doctors comes from both higher administrative levels through weaknesses in the system and from lower levels through the social conditions of the individuals and families affected by HIV.

## Conclusions

Access to financial resources is necessary if PLHIV are to maintain their health by taking ARVs; PLHIV also need to have a set of structures in place to support them when they are on treatment.

The disability grant plays a critical role in supporting PLHIV who are unable to find employment. However, there seems to be some unfairness in the social grant system. We did not find evidence that PLHIV stopped taking ARVs in order to qualify or re-qualify for a disability grant. However, the disability grant is crucial to many PLHIV because it ensures them some degree of economic stability. Given the importance of the public sector ARV roll out, it is decisive for the state to provide these resources.

The traditional understanding of disability implies that the disabled cannot work at all. Being HIV positive and on ARVs represents a position that challenges this kind of understanding. It is possible to work, but the kind of work that PLHIV can do is affected by both HIV and ARVs. This offers scope for further research on the contingencies of living with HIV, on ARVs.

A sound welfare policy could take these more diffuse kinds of living conditions into account, and develop means that are either targeted towards groups with special needs or universal in the sense that all groups in need are covered. Alternatives to disability grants could be a chronic illness grant or a basic income grant, and should be considered by policymakers given the role that social assistance plays in making "positive living" a reality for indigent and unemployed PLHIV.

Furthermore, incentives to create more jobs are pivotal. Given that unemployment is a central concern for many people with HIV, an unemployment grant would also be an important improvement. It is therefore important to ensure a stable economy for unemployed individuals and households affected by HIV through strategic and multi-layered support.

## Competing interests

The authors declare that they have no competing interests, neither financial nor non-financial.

## Authors' contributions

MMDP participated in the data collection, the data analysis and the writing of the paper. She has taken the main lead in the revision of the paper. EAM participated in the data collection, the data analysis and the writing of the paper. ABG participated in the data collection, the data analysis and the writing of the paper. All authors have read and approved the final version of this manuscript.
